# Evaluating the reliability of media reports for gathering information about illegal wildlife trade seizures

**DOI:** 10.7717/peerj.13156

**Published:** 2022-04-05

**Authors:** Kumar Paudel, Amy Hinsley, Diogo Veríssimo, Ej Milner-Gulland

**Affiliations:** 1 Greenhood Nepal, Kathmandu, Bagmati, Nepal; 2 University of Oxford, Oxford, United Kingdom

**Keywords:** Enforcement, Media underreporting, Pangolin, Poaching, Red panda, Rhino, Tiger, Wildlife trafficking, Nepal

## Abstract

Illegal wildlife trade (IWT) is threatening many species across the world. It is important to better understand the scale and characteristics of IWT to inform conservation priorities and actions. However, IWT usually takes place covertly, meaning that the data on species, trade routes and volumes is limited. This means that conservationists often have to rely on publicly available law enforcement reports of seizures as potential indicators of the magnitude and characteristics of IWT. Still, even these data may be difficult to access, leading conservationists to use media reports of seizures instead. This is the case in countries like Nepal, which have limited capacity in data keeping and reporting, and no centralized data management system. Yet reliance on media reports risks introducing further biases, which are rarely acknowledged or discussed. Here we characterize IWT in Nepal by comparing data from three sources of information on IWT between January 2005 and July 2017: seizure reports from three Nepali national daily newspapers, official seizure records for Kathmandu district, and data on additional enforcement efforts against IWT in Nepal. We found a strong positive correlation between the number of official and media-reported seizures over time, but media under-reported seizure numbers, with 78% of seizures going unreported. Seizures of charismatic, protected species were reported more often and seizure reports involving tigers were most likely to be reported (57%). Media reports appeared to be a good indicator of trends and the species being seized but not overall seizure number, with the media largely underestimating total seizure numbers. Therefore, media reports cannot be solely relied upon when it comes to informing conservation decision-making. We recommend that conservationists triangulate different data sources when using seizure data reported in the media to more rigorously characterise IWT.

## Introduction

The illegal wildlife trade (IWT) generates multi-billion-dollar illicit revenues annually, pushing many threatened species towards extinction (UNODC, 2016; UNEP, 2016). In order to design effective interventions, policy-makers and conservation practitioners need information about the scale of IWT, the methods and routes used by traffickers, and the species involved. However, the majority of IWT is hard to observe directly due to its sensitive nature (Duffy, 2016; Barber-Meyer, 2010), meaning that this information is often inferred from proxy sources such as seizure data, sometimes from official records (*e.g.*, Rosen & Smith, 2010), and frequently from media reports (*e.g.*, Siriwat & Nijman, 2018). This means that analyses of IWT often involve implicit assumptions about the reliability of the data being used.

Wildlife seizure data record illegal possession of wildlife apprehended by law enforcement officers. Wildlife seizure data are widely used to infer the trade dynamics, characteristics and flows of IWT (*e.g.*, Hansen et al., 2012; Heinrich et al., 2020; Maheshwari & Niraj, 2018; Paudel et al., 2020; TRACIT, 2019). This information is used to underpin policy decisions such as listing on the appendices of the Convention on International Trade in Endangered Species of Wild Flora and Fauna (CITES), to inform assessments of species’ threatened status in the IUCN Red List, and to prioritise conservation actions (Aryal, 2017).

Due to the difficulties faced in accessing official seizure data, a number of techniques have been developed to monitor news reports on seizures, whether in the hardcopy media or, increasingly, online. These media reports are often used by researchers as a proxy for actual seizure occurrences, where official records are not accessible (*e.g.*, Cheng, Xing & Bonebrake, 2017; Indraswari et al., 2020; Mendis et al., 2021; Morcatty et al., 2020; Nijman, 2015; Siriwat & Nijman, 2018; Verheij, 2019) and form the basis of large databases of seizures hosted by international NGOs (*e.g.*, EIA, 2019; TRAFFIC, 2021). For example, Morcatty et al. (2020) used reported seizure data including from online news to assess wild-cat trade trends in Central and South America; Siriwat & Nijman (2018) analysed seizure records from online media to identify spatial and temporal patterns of Siamese rosewood; Heinrich, Koehncke & Shepherd (2019) analysed the pangolin trade volumes and routes using seizure records including from German media. Media reports are also used in the study of human-wildlife conflict (Hughes et al., 2020). As well as use by researchers, the media plays a key role in influencing which issues are seen as important by the public and policymakers. Media-reported seizures show only the cases which are accessible to the media, as well as reflecting the issues they are interested in publishing about (Hansen et al., 2012). The use of media reports to make inferences about wildlife trade, whether by researchers, policymakers or the public, therefore introduces another layer of bias over and above those from the seizure process itself. The accuracy of media reports of IWT seizures is unknown but media underreporting of other crimes has been shown to vary widely, from 7% for law-enforcement related deaths in the USA (Feldman et al., 2017), to 76% for homicides in Iraq (Siegler et al., 2008) and 98.5% for chemical accidents in Florida (Lynch, Stretesky & Hammond, 2000).

However, despite their importance, biases in seizure data are often not well understood by researchers, policymakers and others who might use these data to draw conclusions on trade. These biases emerge for many reasons, for example due to variations in detectability of IWT, the relationship of seizure rate to enforcement effort, differences between countries and agencies in their capacity to collect the information, and their recording and reporting practices (Table 1). In addition, certain species may suffer from an additional form of reporting bias linked to the difficulty in identifying different species. For example, specific species of plants, reptiles and birds are comparatively harder to identify than tigers, leading to their underrepresentation in seizure records. Enforcement efforts may also have some operational biases, for example towards certain priority geographical areas, or particularly charismatic or high-profile species. These biases are challenging to quantify and control for (Dobson et al., 2020; Underwood, Burn & Milliken, 2013; Keane, Jones & Milner-Gulland, 2011). One of the only examples of systematic monitoring of IWT seizures is the Elephant Trade Information System (ETIS), which is used to inform CITES actions on the ivory trade (CITES, 2004). The ETIS has a sophisticated modelling process to account for the biases in seizure data (Underwood, Burn & Milliken, 2013). This involves using a Bayesian hierarchical model to correct for seizure and reporting rates by identifying proxy variables that are associated with variation in these rates over time and between countries (Underwood, Burn & Milliken, 2013).

**Table 1:** Potential biases that we hypothesise are likely to exist in each source of data used in the study (shaded cells represent biases that cannot be investigated in this study but should still be considered). We define bias as an underrepresentation or overrepresentation in different sources of data of particular types of wildlife trade products or seizures.

**Type of data**	**Potential sources of bias**	**Potentially overrepresented**	**Potentially underrepresented**
Official seizure records	Enforcement officers may find certain species/products easier to detect or identify, or may be focused on species that are higher priority due to protection status or financial value. Conversely some high profile species seizures may not be officially recorded by the authorities to maintain the image of enforcement effectiveness.	Charismatic mammals; easily detected IWT products; valuable IWT products	Plants; animals that are not protected/high profile; species that are traded as processed products; potentially some very high profile species.
Media reports	Editors may favor stories about species they think will be of interest to readers, or seizures that are out of the ordinary in some way due to volume of products seized/financial value, or species involved. Seizures from cities may be picked up more, and more recent seizures may be overrepresented as illegal wildlife trade has risen in prominence.	Charismatic mammals; larger seizures; multiple arrestees; valuable seizures; big cities; more recent seizures	Small seizures; plants/non-charismatic animals; seizures that take place in rural areas; older seizures; lesser known species
Police records of enforcement operations	Officers may be more likely to focus on high-value species or seizures that are linked to other crimes. Seizures may be focused on urban areas with more police, or priority areas where more operations take place.	High-profile species; data on their own operations; the areas where they operate	Seizures made during other enforcement operations; areas outside their jurisdiction

While studies into how the media perceive IWT have been carried out (*e.g.*, Gao et al., 2016), little is known about how dominant media narratives can influence public perceptions of the wildlife trade. Furthermore, there is little information about public perception of IWT and conservation issues in Nepal, except community forestry and conservation projects (Mehta & Kellert, 1998; Müller-Böker & Kollmair, 2000; Acharya, Petheram & Reid, 2004). It is possible that biases in media-reporting towards certain species (*e.g.*, the focus on trade in species such as elephants and pangolins, relative to plants) could influence public opinion and cause prioritisation of one taxonomic group over another (Hansen et al., 2012). There is therefore an urgent need to better understand the biases in seizure news reports in conservation, to address their potential to distort public opinion, policy processes and wildlife conservation strategies.

Although policy-makers may have access to official records in many countries, a reliance on media reports is often found in countries with limited capacity to compile official seizure databases, including in many IWT hotspots such as Nepal. Nepal is one of the richest countries for biodiversity and hosts many of the world’s threatened species targeted by poachers, including royal bengal tiger (*Panthera tigris tigris*), greater one-horned rhinoceros (*Rhinoceros unicornis*), red panda (*Ailurus fulgens*), bears (*Ursus arctos, U. thibetanus* and *Melursus ursinu* s), pangolins (*Manis pentadactyla* and *M. crassicaudata*), leopards (*Panthera uncia, P. pardus, Neofelis nebulosa*). It is also well connected to India and to one of the biggest markets for wildlife derivatives, China (DNPWC, 2017; Wong, 2019). As an important source and transit country for IWT within South Asia, there has been a growing conservation interest in Nepal in combating IWT, and IWT seizures influence national-level priorities for action. However, outside of large cities such as Kathmandu, official data on seizure cases are registered in local forest offices in paper files, and recorded in Nepali. Collection of official seizure data is difficult without travelling to these rural offices and manually sorting through bundles of registers. The media access seizure reports through press conferences, requests to the authorities from reporters, and direct contact with officials.

The media has been described as the fourth pillar of modern society (Kaur, 2017), and it is clear that, in Nepal, newspapers are an important source of information for decision makers (Internews, 2011), often influencing conservation policy (Sunam, Bishwokarma & Darjee, 2015). Newspaper-reported wildlife seizures have been described as “*directly influencing the plans and actions of the government and its concerned departments. In the country like Nepal which has limited research capacity, we are more dependent on the media to grasp the information and inform conservation priorities*” (FR Kharel, pers. comm., former director general of Department of National Parks and Wildlife Conservation, 07 August 2019). Therefore, news reports of seizures play an important role in decision making in Nepal, and inform both the general public and policy makers about the scale and characteristics of IWT in the country. Thus, a better understanding of seizure news reports and their biases will be very useful to shape actions to combat IWT.

Nepal provides an ideal study site to understand the use of media reports in prioritizing actions to control illegal wildlife trade and conservation interventions; as it is an important source and transit country for illegal wildlife trade in South Asia (Paudel et al., 2020) with limited enforcement capacity and no centralized IWT database. Here, we use official seizure records gathered from the local Division Forest Office (DFO) in Kathmandu, Nepal and police records of enforcement operations, to verify reports of IWT seizures from three popular Nepalese newspapers, controlling for changes in levels of official enforcement effort. We aim to understand the factors that affect biases in reporting of seizures in these three newspapers and to better understand the biases that come with different data sources, defined as an overrepresentation or underrepresentation of specific types of products or wildlife trade activities in these data sources (Table 1). Further, we are looking at both reporting bias (ie chance of being reported as a function of the media outlet) and how that bias varies with respect to different characteristics of the event (*e.g.*, species, size of the seizure and location). We also aim to investigate how triangulation using multiple data sources may help to overcome these biases.

## Materials & Methods

### Seizure news reports

A team of four researchers led by corresponding author of the paper reviewed the contents of three Nepali national dailies, *Gorkhapatra, Kantipur* and *The Kathmandu Post*, from January 2005 to July 2017, at Kantipur Publication House’s library and the Central Library of Tribhuvan University. We selected the most widely read newspapers in Nepal whose archives are available. All three are daily newspapers; Gorkhapatra is the official newspaper of the Government of Nepal, Kantipur is one of the most popular and widely subscribed-to privately owned Nepali dailies, and The Kathmandu Post is a privately owned English-language daily published by Kantipur Media Group. We did not review online media since the archive of the local news portals was not available for this time frame.

The team developed a protocol to ensure that seizure reports were found in each newspaper and that biases were reduced. The protocol included putting newspaper on a flat table and skimming all pages looking for wildlife related headlines and images. We then checked the contents of wildlife-related news to differentiate seizure news from other wildlife-related news *e.g.*, human-wildlife conflicts, general conservation news. To minimise the chance that any reports would be missed, the researchers double-checked every page of the print-copies of each newspaper, and scanned any wildlife seizure news reports found, transcribing all the information related to species, amount, location, arrestee, origin and destination into a spreadsheet. We collected data on any reported wildlife seizure occurring in any location in Nepal. Despite this protocol, it is possible that some reports could have been missed. In situations where newspapers reported seizures of unknown wildlife parts, they were categorised as ‘Others’.

The reports were cross-referenced between the newspapers, so that the number of reports of the same incident in different newspapers could be analysed. There were situations where different newspapers published the same seizure case on different dates and similar seizures appeared in multiple newspapers. Reports were taken to be of the same incident based on key details including the date, amount, species composition, location and name of the arrestees. These key facts were found to be always consistent when similar seizures were found.

### Official seizure records and enforcement effort

Generally, Nepal Police, Customs, Armed Police Force, Divisional Forest Office (DFO) and Protected Area (PA) authorities detect and seize illegal wildlife consignments and then hand over the case to the DFO or PA authorities for further prosecution. These authorities then forward cases to the District Court. In principle, records of all IWT seizures exist in paper registers in different places. Only a few offices have developed a database of these IWT seizures, one of which is Kathmandu District Forest Office. We obtained the official IWT seizure records of Kathmandu district from January 2005 to July 2017.

In recent years, IWT seizure cases are growing, as is enforcement effort. Before 2010 all the IWT investigations and operations were carried out by Nepal Police and DFO and PA authorities. To respond to the growing IWT threat, in 2010 Nepal’s police force founded a special Wildlife Crime Control wing of the Central Investigation Bureau (CIB) to deal with wildlife trade-related investigations. After that, most of the IWT seizures were carried out by that wing of CIB. Therefore we also collected data on the efforts of the CIB over time, including the size of the CIB’s wildlife crime control unit and the number of undercover operations carried out per year across Nepal.

All official datasets were provided by official sources and we also reviewed their annual reports, newsletters, official website records and press releases. Further, we reached out to the CIB and DFO to seek permission to publish the data and we were advised in writing that no further permission was needed.

### Data analysis

We initially analysed the data using basic descriptive statistics such as species, volume of seizure, location, number of arrestees *etc*. to characterise patterns of reporting across time and space. We excluded 2017 from the trend analysis as we did not have a full dataset for that year. We used Spearman’s rank correlation to look for general relationships between the number of unreported and reported seizures and two-tailed Fisher’s exact test, based on 100,000 Monte Carlo simulations, to look for differences in reporting rates between species or species groups. We then used binomial logistic regressions to better understand the characteristics of a seizure that are linked to an increased probability of reporting such as protection level, year, number arrested, species. Lastly we used piecewise regression to better understand potential changes in number of seizures across time. Statistical analyses were conducted using R 3.3.2 (R Core Team, 2013), using core packages as well as dpseg, and IBM SPSS 25. A summary of the variables used and their coding is included in Table 2.

**Table 2:** Variables used in regression analysis to understand the drivers of wildlife seizure reporting in three Nepali newspapers (The Kathmandu Post, Kantipur and Gorkhapatra).

Variables	Description	Coding
Protection Level	Level of legal protection given to the species seized. When species belonging to different legal categories were seized together, the record was assigned the highest level of protection as per National Parks and Wildlife Conservation Act.	(0) not protected, (1) level 1 legal protection, (2) level 2 legal protection, according to Nepal’s National Parks and Wildlife Conservation Act^a^
Year	Calendar year in which the seizure took place	Year number in Gregorian calendar
Number arrested	Number of people arrested	Number
Species dummy coded for each of: Elephant, Tiger, Rhino, Musk deer, Red panda, Pangolin, Leopard Bear, Owl, Others^b^	Presence or absence of specific species products in a seizure	(0) Absence, (1) Presence

**Notes.**

a Level 0: protected species with sanctions of a fine up to NPR 20,000 or 6 months imprisonment or both; Level 1: protected species with sanctions of fine NPR 100,000 to 500,000 or 5 years to 10 years imprisonment or both; Level 2: protected species with higher sanctions of fine NPR 500,000 to 1,000,000 or 5 years to 15 years imprisonment or both, this includes one-horned rhinoceros, tiger, elephant, musk deer, clouded leopard, snow leopard and gaur.

b Presence or absence of wildlife products in a seizure from species that are less frequently seen in trade (*e.g.*, seahorse, wild boar, spotted deer and turtle, otter, python).

## Results

While analyzing the wildlife seizure reports, temporal trends are important for understanding changes over time given the evolving geopolitical and economic context. Potential drivers of trends include changes in the prioritisation of enforcement efforts, economic growth, accessibility of areas containing tradable species, technological advances such as tracking devices, major political events, natural disasters, policy changes *etc*. IWT incidence is also likely to depend on spatial factors such as proximity to international borders, protected areas, species distribution and business hubs (see ROUTES, 2019). All of this is affected by the dependence of seizure occurrence on the level of the enforcement effort, and so it is key to understand if and how this effort has varied. The number of enforcement operations increased year on year between 2011 (*n* = 18) and 2017 (*n* = 50 until July; Table 3). The number of people involved in CIB wildlife crime control operations increased from seven in 2011 to 42 in 2013 and remained stable after that. This increase in operations is due to the expansion of focus to other regions beyond Kathmandu from 2013.

**Table 3:** Number of wildlife crime control operations conducted across Nepal by the Central Investigation Bureau (CIB) of the Nepal Police, size of CIB team and CIB-led wildlife seizures in Kathmandu district between 2011 and 2016.

Year	Number of operations across Nepal	Number of people in CIB wildlife crime control team	Total number of seizures in Kathmandu
2011	18	7	16
2012	25	12	36
2013	29	42	30
2014	34	42	42
2015	52	42	37
2016	58	42	49
Total	216	NA	210

### National wildlife seizure media reports

**Figure 1:**
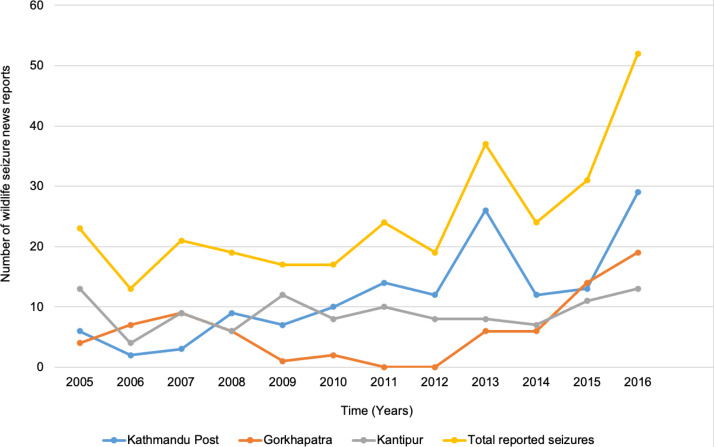
Number of wildlife seizure news reported in The Kathmandu Post, Gorkhapatra and Kantipur dailies during 2005 to July 2016.

Between January 2005 and July 2017, a total of 317 reports on wildlife seizures were found in the print versions of the three major daily newspapers; *The Kathmandu Post, Gorkhapatra* and *Kantipur*, ranging from 13 in 2006 to 52 in 2016 (Fig. 1). The percentage change in the number of seizures reported year on year was highly variable across this period (from −77% to +62%; Table S7). Different newspapers reported different seizures, with no single newspaper reporting all seizures in any year.

**Figure 2:**
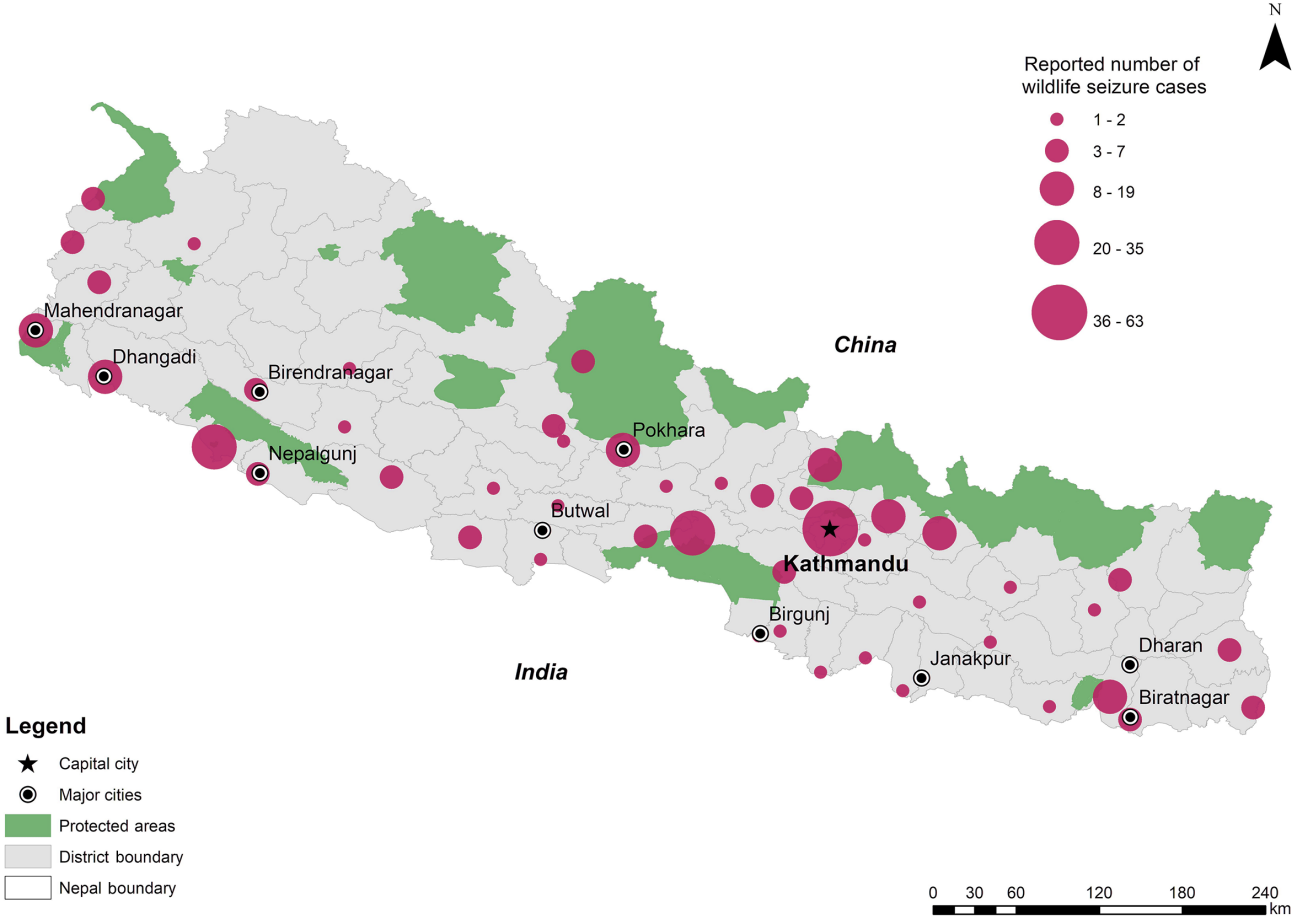
Number of wildlife seizure news reports from different districts published in The Kathmandu Post, Gorkhapatra and Kantipur dailies during Jan 2005 to July 2017 Number of wildlife seizure news reports from different districts in the newspapers varies from 0 to 63. Due to these variations, equal intervals could not project the data clearly, so we choose represent them in an ordinal scale.

The highest number of reports by any one newspaper was in The Kathmandu Post in 2016 (*n* = 29), which was reflected in similar (but smaller) peaks in Kantipur (*n* = 18) and Gorkhapatra (*n* = 12). Out of the 317 seizures, five were reported in all three newspapers and 26 reported in at least two newspapers. Most of the seizures reported in multiple newspapers were sourced from Rastriya Samachar Samiti (National News Agency) and official press releases of Protected Area officials, Nepal Police and Division Forest Offices. However, the value of the seized wildlife was reported differently between newspapers in some seizure cases (*n* = 5). These cases involved the seizure of pangolin, tiger, rhino and leopard specimens. For example, The Kathmandu Post mentioned the reported value of a leopard skin seized on 10 March 2016 as Nepalese Rupee (NPR) 30000 while other reported NPR 80000 for the same skin. In terms of overall trend, there was a steady increase over time (F(1, 149) = 19.84, *p* < 0.001, *R*^2^ = 0.11), with piecewise linear regression finding a positive slope (0.02) but no breakpoints when looking at the number of monthly reported seizures across the study period.

Seizure cases from 49 districts were reported (Fig. 2), but 52% of the seizures were reported from only five districts: Kathmandu, Chitwan, Bardiya, Kanchanpur and Kailali. These districts have one key common characteristic; they are either near protected areas or host a regional trade hub. No reports were found from 26 districts, most of which are geographically remote, hilly and mountainous districts. There could be differences in media coverage in certain districts impacting the coverage especially due to the absence of local staff journalists; however, this information was not available.

### Official seizures and media-reported seizures in Kathmandu

The total number of wildlife seizures in Kathmandu district varied across years (Fig. S1), with an increasing trend over time. Of the 289 seizures in Kathmandu district, 78% (*n* = 226) were not reported by any newspaper. Despite a significant correlation between total and reported number of seizures (*R*_*s*_ (13) = 0.866, *p* < 0.001) the proportion of seizures reported varied considerably between years. In 2006 only 9% of seizures were reported while 38% of seizures were reported in 2011.

**Figure 3:**
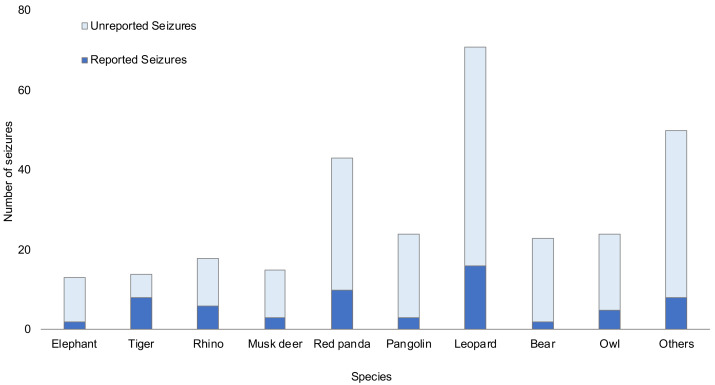
Number of reported and unreported wildlife seizures per species. Reported seizures refer to a report being found in any of the three newspapers.

Unreported seizures therefore made up the majority of seizure events for all groups except for tigers, for which 57% of seizures were reported in the media (Fig. 3). The proportion of reported seizures did not differ across species (*p* = 0.087, Fisher’s exact test) when considering the three newspapers altogether. However, when considering them separately, there were differences between species in coverage by The Kathmandu Post (*p* = 0.039, Fisher’s exact test), where an analysis of standardised residuals showed that rhinos and tigers were disproportionately reported on. These differences were not found for Gorkhapatra (*p* = 0.062, Fisher’s exact test) or Kantipur (*p* = 0.529, Fisher’s exact test). The taxa with the highest proportion of reported seizures were tiger (57%), rhino (33%), red panda (23%) and leopard (23%), whereas bears (9%), pangolin (13%) and elephant (15%) had the lowest rates.

The number of reported seizures was evenly distributed among newspapers (*χ*^2^(2, 63) = 2.57, *p* = 0.277). We found no statistically significant relationship between the level of protection of the species seized, the number of arrestees, or the year of seizure, and the probability of a seizure being reported in any of the newspapers separately (Tables S1–S3) or when considered jointly (Table S4).

## Discussion

Our study demonstrates that, in Nepal, the media significantly underreports wildlife seizures, with 78% of wildlife seizures in Kathmandu district not reported in any major newspapers. Kathmandu may not be representative of the entire country but it can provide important insights as it is the capital city of Nepal, a popular wildlife trade transit route, and a known hotspot for some types of wildlife seizures (Paudel et al., 2020; Wong, 2019; Dangol, 2015). In our study, under-reporting appeared not to be biased either between newspapers or according to species specific factors, even though we found taxonomic bias in reporting for one out of the three newspapers. The reporting biases of a newspaper, and the variation in these biases, could be due to reasons including the editors and reporters having an interest in wildlife trade or a particularly good access to information on seizures, increasing seizure events of certain species, more conservation attention on these species than others that raised their profile and other competing news stories. Aggregating reports from multiple media sources, in our case three difference newspapers, reduces under-reporting, but still a large proportion of the seizures in Kathmandu went unreported. Our results support calls to recognise the importance of triangulating data from different sources to draw conclusions about complex conservation issues, especially where these data will be used to set global or national conservation priorities (Booth et al., 2020; Dobson et al., 2020). Dietrich & Eck (2020) make a similar call to integrate information from media sources with information derived from other sources, with respect to a different issue—reporting of events of political violence.

Media reports are likely to remain important tools for drawing inferences about some aspects of IWT, but our findings of significant media-underreporting of seizures suggest that they should not be used as a proxy for the overall scale of IWT. Reporting is also likely to vary by location, over time and in different contexts, as demonstrated by studies of media under-reporting examples focused on different crimes (*e.g.*, Lynch, Stretesky & Hammond, 2000; Siegler et al., 2008; Feldman et al., 2017; Dietrich & Eck, 2020). The steady increase in the number of reported seizures over time could be linked to increasing conservation awareness, enforcement effort (with CIB increasing the number of operations every year since its inception in 2011) and reporting efforts by local authorities, rather than representing an actual increase in the underlying level of IWT in Nepal.

The use of official seizure data to draw high-level conclusions on wildlife trade is likely to increase now that international illegal trade reporting is mandatory for CITES Parties (CITES Res. Conf 11.17 (Rev. CoP17)). These data will be made available for research and analysis of wildlife crime by the United Nations Office on Drugs and Crime (UNODC), but it is not clear whether they will be made public. CITES data also have caveats, particularly in terms of reporting. There are many discrepancies between exporter-reported and importer-reported quantities with respect to the same trade events. Further, often the traded species and units are not very clear (Robinson & Sinovas, 2018). As a further example of the issues with CITES data, in Democratic Republic of Congo between 2014 and 2017, 13.4% of the trade in wild grey parrots reported on social media was not found in CITES trade records (Martin, Senni & D’Cruze, 2018).

All these issues mean that other sources of information about seizures (including the media) are likely to remain important for researchers, decision-makers, and practitioners for some time. This is particularly true when official seizure reports are difficult to access, either because they often contain sensitive information, or because they are not centrally collated into a usable database. The latter is the case in Nepal, where seizure records are stored in paper files at local Division Forest Office and protected area offices in the regions where the seizure occurred, and where media reports of wildlife trade seizures are an important source of information for the government (FR Kharel, pers. comm., former director general of Department of National Parks and Wildlife Conservation, 07 August 2019). With this ongoing importance of media-reported seizure data, our findings show that biases in these data, and the scale of underreporting, must be carefully considered. This is important because Nepal is a biodiversity hotspot, and one which contains a number of species in trade, and increasing demand for the illegal wildlife trade. It is also an important transit route between South Asian countries and China, and the markets of south-east Asia, meaning that effective law enforcement has a wider regional impact (Wong, 2019).

While media reports should not be used to infer the scale of seizures, we show that, in the case of Nepal, using media-reported seizures would lead to appropriate inferences about the overall trend in seizures and the general nature of seizures. While more than half of tiger seizures were reported, other species were much less likely to be reported. The Kathmandu Post matched our hypotheses on taxonomic reporting biases, considering all three newspapers in aggregate removed these significant biases. For example, English-language newspapers may be more or less interested in IWT, or interested in different species, than local language newspapers. These biases must be considered, as there is evidence that media saliency can play a role in policy-makers’ decisions (Weaver, 1991), meaning that biases in reporting of issues that are politically sensitive, such as the illegal wildlife trade, may have much wider implications. For example, based on media reports, Nepal has prioritized training enforcement to track trade in tiger parts and rhino horn, because there is the perception that these are the most traded species. Yet, there are many another wildlife products, such as orchids and medicinal plants, that are traded in large volumes (Subedi et al., 2013; Pyakurel et al., 2019), but are not prioritized. In addition to influencing policy, these reporting biases can also have far-reaching consequences for conservation in other ways, as there is evidence that media coverage directly influences the public’s knowledge and views on issues (McCombs & Shaw, 1972). Further, the media has the potential to influence prioritisation of different conservation issues amongst the public, funders and scientists (Veríssimo et al., 2014). Therefore, underreporting by the media may mean that global attention towards certain species or areas is not as high as it should be, with the concomitant underinvestment in funding for conservation and political support to the government departments responsible for controlling IWT. For rhinos and tigers this may mean less funding than is needed, but for taxa that are less likely to be seized in the first place (*e.g.*, medicinal plants: He et al., 2018), a reliance on reported seizures to inform policy could lead to serious gaps in attention.

We show how traditional media sources report wildlife trade seizures, but it is likely that the move of media into online channels (Newman et al., 2019), and the ongoing deprofessionalisation of global media following a shift to social media, will change these biases (Splichal & Dahlgren, 2016). For example, the competition for page-space or time which affects print and broadcast media may loosen, such that more seizure reports may be placed online. However it may also be that a reduction in professional staff may reduce the investigative focus on particular issues, such that even if more material is present, the prominence of particular items in online media may be driven more by social media trends and public concern than a newspaper’s (or government’s) priorities. Further work is needed to understand potential underreporting biases of wildlife trade seizures in these ‘new’ media sources, especially as accuracy and biases in reporting have been shown to vary greatly between online platforms (Baly et al., 2018).

Despite some drawbacks in their use, there is the potential for further work to look at how media reports could be used to gather information on the underlying seizure data, and validate reporting accuracy. This will likely be rendered easier in the future as newspapers move online and the methods needed to collect and analyze these kinds of datasets, the focus of the field of conservation culturomics, continue to evolve (Correia et al., 2021). It is known that some seizures are not reported up to the national level, and even those that involve protected species at border crossings may not be reported to official global databases such as those operated by CITES, whether through lack of capacity or depending on the country’s priorities, commitments, and inter-agency collaborations (Underwood, Burn & Milliken, 2013). Our study reviewed both seizure and official reports of one district, but where large datasets of both seizures and media reports exist, there may be scope to expand our analysis and develop techniques to estimate the number of seizure occurrences from media reports. This could be based on existing methods such as occupancy or capture-mark-recapture modelling, which have been used to quantify media underreporting of law-enforcement-related deaths (Feldman et al., 2017), as well as to estimate the scale of IWT on online platforms (Yeo, McCrea & Roberts, 2017), and physical wildlife markets (Lam, 2012). Therefore, the application of these methods to media reports of wildlife seizure data could allow conservationists to use freely available information to estimate the scale of seizures, although the assumptions underlying these types of analyses may not be met for all IWT markets (Lam, 2012). Another approach would be to extend the use of complex Bayesian hierarchical models, which have been applied to correct for biases in official seizure reports of ivory (Underwood, Burn & Milliken, 2013) to understand biases in media reports.

In our research, only a few news reports mentioned the origin and destination of the seized wildlife parts. Thus, we did not collect information about routes in this study. However, this could be of interest to further research. While, at least in the medium term, there is still an important role for old-fashioned detective work to find and document official seizure records, media-reported seizure data can be used in some cases, and with further work to understand and address biases it will become more useful in the future. These biases can be reduced by developing inter-agency data-sharing mechanisms, triangulating the data from different sources, developing centralized databases and better networking between media, data centers, researchers and policy makers directly. We suggest triangulating data from media reported seizures with other sources and highlight the importance of being aware of the biases this data carries while interpreting trends.

## Supplemental Information

10.7717/peerj.13156/supp-1 Supplemental Information 1 Detailed statistical results, total reported seizures in the newspapers including species list

10.7717/peerj.13156/supp-2 Supplemental Information 2 Nepali newspapers (The Kathmandu Post, Kantipur and Gorkhapatra) reported illegal wildlife seizures in Kathmandu including seized species, their protection level in law and arrestee size See Table 2 for the details of the variables

10.7717/peerj.13156/supp-3 Supplemental Information 3 R scripts

## Additional Information and Declarations

### Data Availability

The following information was supplied regarding data availability: The raw and working data are available in the Supplementary Files.
