# Supplementary material for: Evaluating the reliability of media reports for gathering information about illegal wildlife trade seizures
Source: PeerJ. 2022 Apr 5;10:e13156. doi: 10.7717/peerj.13156 (PMC8992658; doi:10.7717/peerj.13156)
Supplement: Supplemental Information 1 [file peerj-10-13156-s001.docx]

**Supplementary Materials**

**Table S1** Output of regression model of variables associated with reporting of wildlife seizures in the newspaper The Kathmandu Post

| **Variable** | **Coefficient** | **Standard Error** | **p** |
| --- | --- | --- | --- |
| (Intercept) | 62.75 | 135.68 | 0.637 |
| Arrestees | 0.202 | 0.268 | 0.45 |
| Protection level | 0.459 | 0.278 | 0.099 |
| Year | -0.033 | 0.067 | 0.628 |

**Table S2** Output of regression model of variables associated with reporting of wildlife seizures in the Nepalese newspaper Kantipur

| **Variable** | **Coefficient** | **Standard Error** | **p** |
| --- | --- | --- | --- |
| (Intercept) | 62.426 | 136.922 | 0.648 |
| Arrestees | 0.285 | 0.265 | 0.282 |
| Protection level | 0.245 | 0.276 | 0.376 |
| Year | -0.032 | 0.068 | 0.634 |

**Table S3** Output of regression model of variables associated with reporting of wildlife seizures in the Nepalese newspaper Gorkhapatra

| **Variable** | **Coefficient** | **Standard Error** | ***p*** |
| --- | --- | --- | --- |
| (Intercept) | -0.08 | 0.03 | **0.009** |
| Arrestees | -0.008 | 0.3464 | 0.981 |
| Protection level | 0.226 | 0.381 | 0.554 |
| Year | 0.395 | 0.151 | **0.009** |

**Table S4** Output of regression model of drivers of reporting of wildlife seizures in three Nepalese newspapers (The Kathmandu Post, Kantipur and Gorkhapatra)

| **Variable** | **Coefficient** | **Standard Error** | ***p*** |
| --- | --- | --- | --- |
| Intercept | -50.282 | 97.168 | 0.605 |
| Number arrested | 0.155 | 0.183 | 0.398 |
| Protection level | 0.371 | 0.191 | 0.052 |
| Year | 0.024 | 0.048 | 0.617 |

* p < 0.05; ** p < 0.01.

**Table S5**  - Results of chi-square goodness-of-fit test, based on 10 000 Monte Carlo simulations, on media reported versus actual wildlife seizures.

| **Media source** | **Chi square coefficient** | **Sample size** | ***p*** |
| --- | --- | --- | --- |
| The Kathmandu Post | 16.902 | 25 | **0.048** |
| Gorkhapatra | 11.719 | 15 | 0.215 |
| Kantipur | 6.717 | 25 | 0.668 |
| The Kathmandu Post + Gorkhapatra + Kantipur | 12.314 | 63 | 0.192 |

**Table S6** List of the species and number of seizures in Kathmandu from January 2005 to July 2017

| **Species** | **IUCN Red List Categories** | **Number of seizures** |
| --- | --- | --- |
| Asian Elephant | Endangered | 13 |
| Tiger | Endangered | 14 |
| Greater One horned Rhino | Vulnerable | 18 |
| Musk deer | Endangered | 15 |
| Red panda | Endangered | 43 |
| Pangolin | Endangered | 24 |
| Common leopard | Vulnerable | 71 |
| Asiatic Black Bear | Vulnerable | 23 |
| Owl sp. | Least Concern to Endangered | 24 |
| Barking deer | Least concern | 9 |
| Unidentified birds | NA | 7 |
| Unknown wildlife parts | NA | 6 |
| Spotted linsang | Least concern | 4 |
| Python | Vulnerable | 3 |
| Seahorse sp. | Least Concern to Endangered | 3 |
| Otter sp. | Near Threatened to Endangered | 3 |
| Spotted deer | Least concern | 4 |
| Wild cat sp. | Least Concern to Endangered | 2 |
| Turtle/tortoise sp. | Least Concern to Endangered | 3 |
| Red giant flying squirrel | Least concern | 2 |
| Tibetan antelope | Endangered | 2 |
| Peacock | Least concern | 1 |
| Clouded leopard | Vulnerable | 1 |
| Monitor lizard sp. | Least Concern to Endangered | 1 |
| Mugger crocodile | Vulnerable | 1 |
| Wild boar | Least Concern | 1 |
| Rabbit sp. | Least Concern to Endangered | 1 |
| Unknown snake | NA | 1 |

Table S7: Total reported seizures in three newspapers and percent change in the reporting

| Year | Total reported seizure | Percent change in reporting each year | Percent change in reporting (baseline year 2005) |
| --- | --- | --- | --- |
| 2005 | 23 | NA | NA |
| 2006 | 13 | -77 | -77 |
| 2007 | 21 | 62 | -10 |
| 2008 | 19 | -11 | -21 |
| 2009 | 17 | -12 | -35 |
| 2010 | 17 | 0 | -35 |
| 2011 | 24 | 41 | 4 |
| 2012 | 19 | -26 | -21 |
| 2013 | 37 | 49 | 38 |
| 2014 | 24 | -54 | 4 |
| 2015 | 31 | 23 | 26 |
| 2016 | 52 | 40 | 56 |


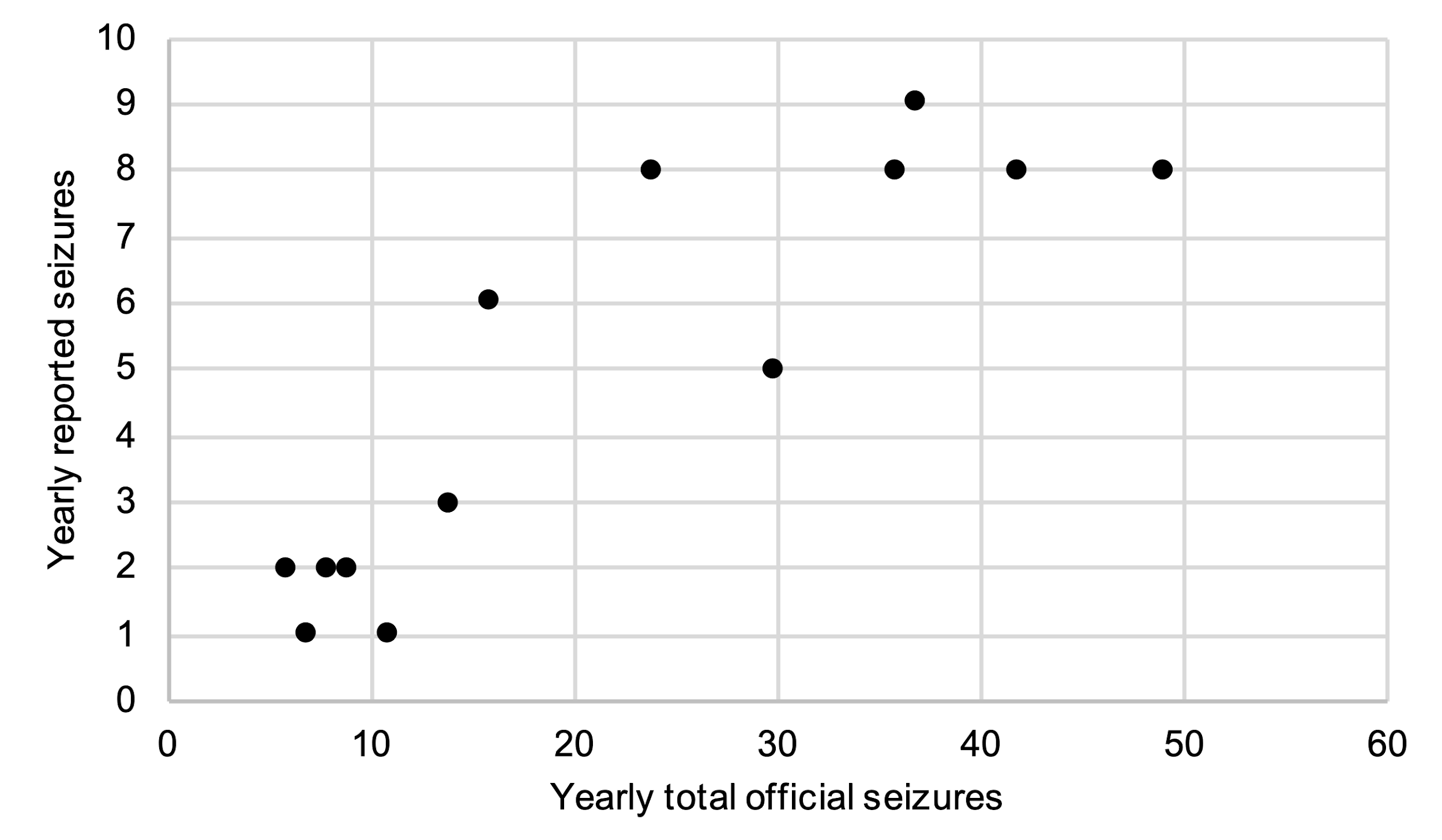


**Figure S1.** Yearly number of wildlife seizures in total and as reported by three newspapers in Nepal (The Kathmandu Post, Kantipur and Gorkhapatra) for Kathmandu district only. Data from 2017 corresponds only to the first seven months of the year.

**
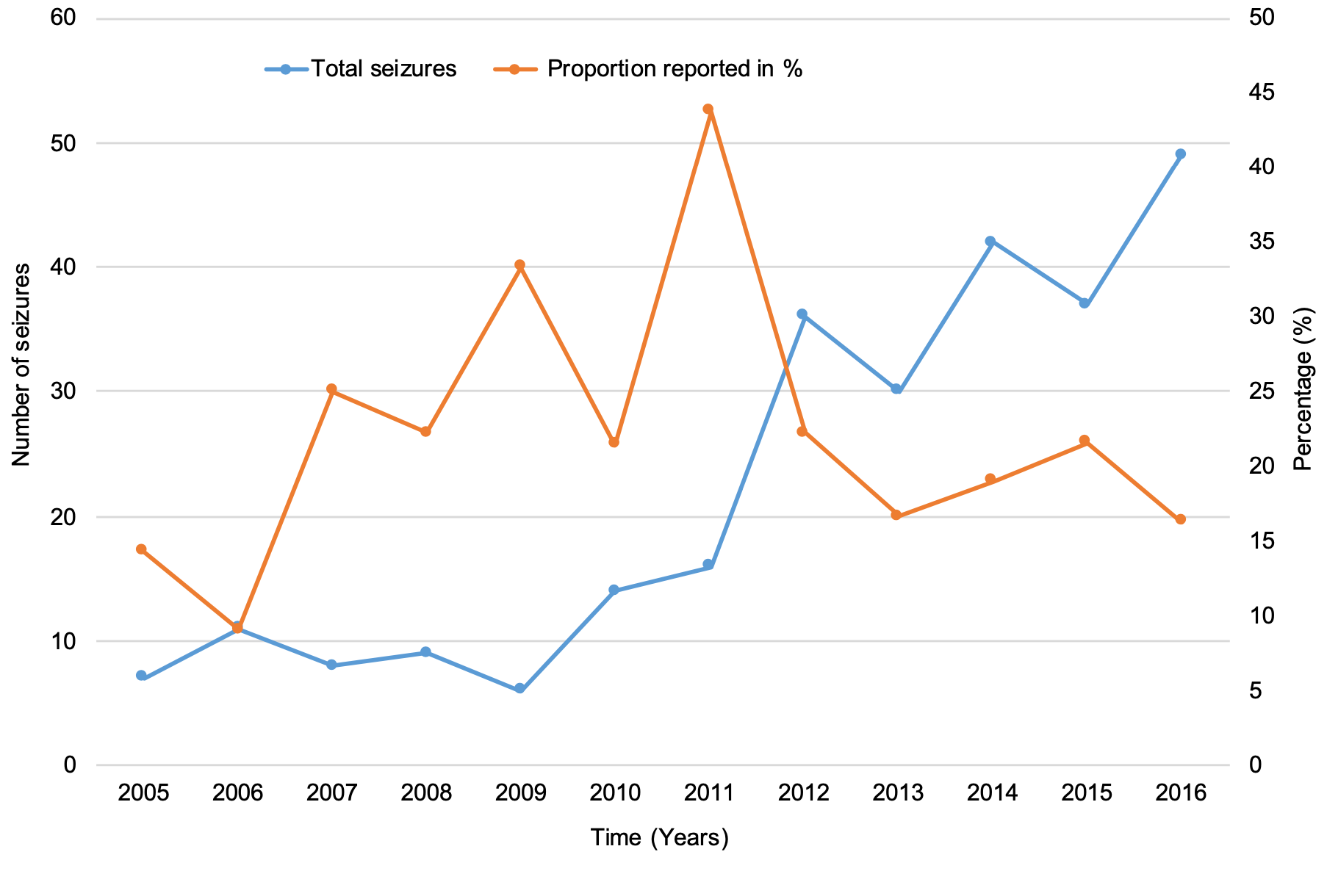
**

**Figure S2:** Yearly number of wildlife seizures in total and proportion reported by three Nepali newspapers (The Kathmandu Post, Kantipur and Gorkhapatra) for Kathmandu district only.


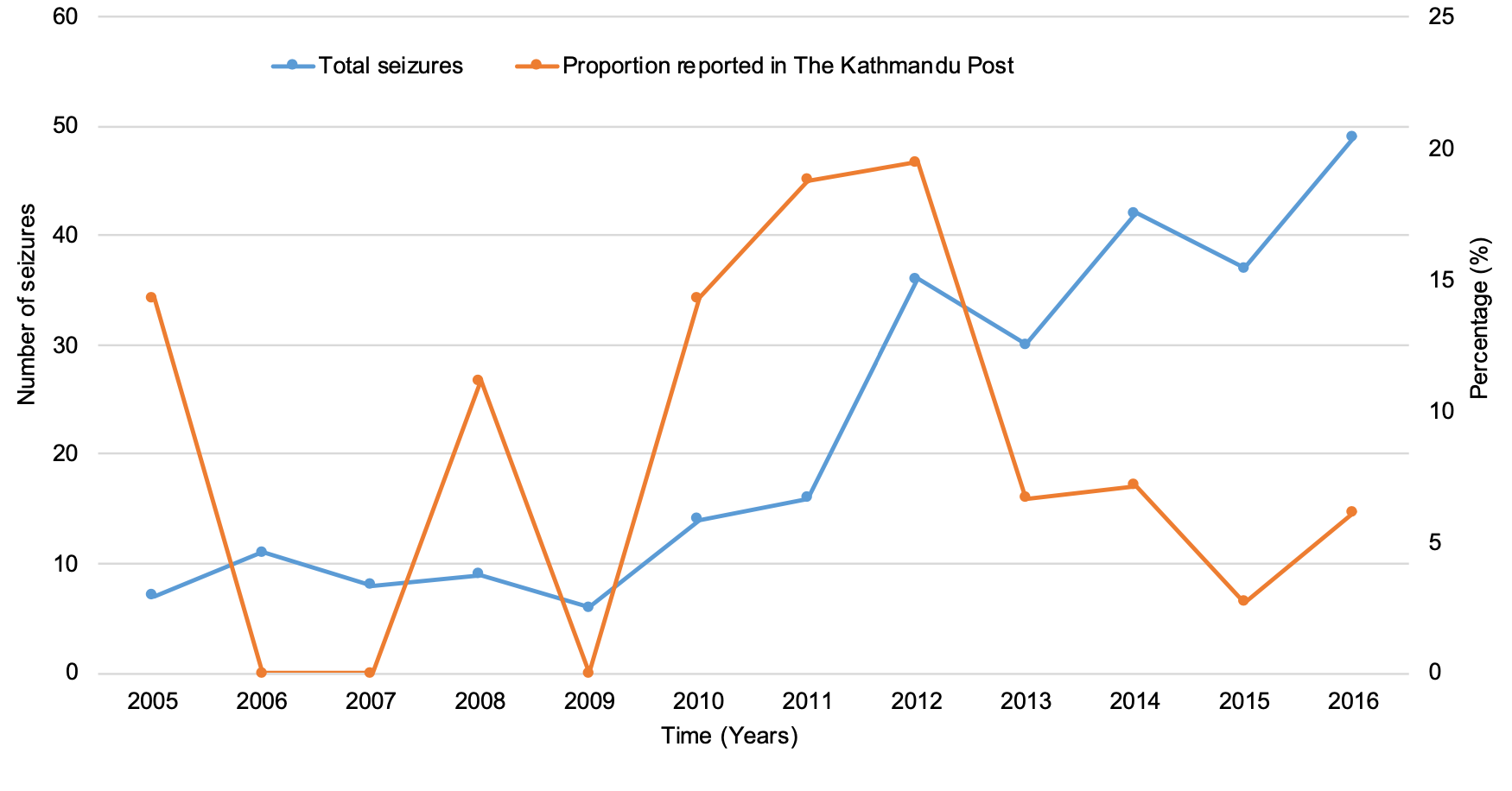
**Figure S3:** Yearly number of wildlife seizures in total and proportion reported by The Kathmandu Post for Kathmandu district only.

**
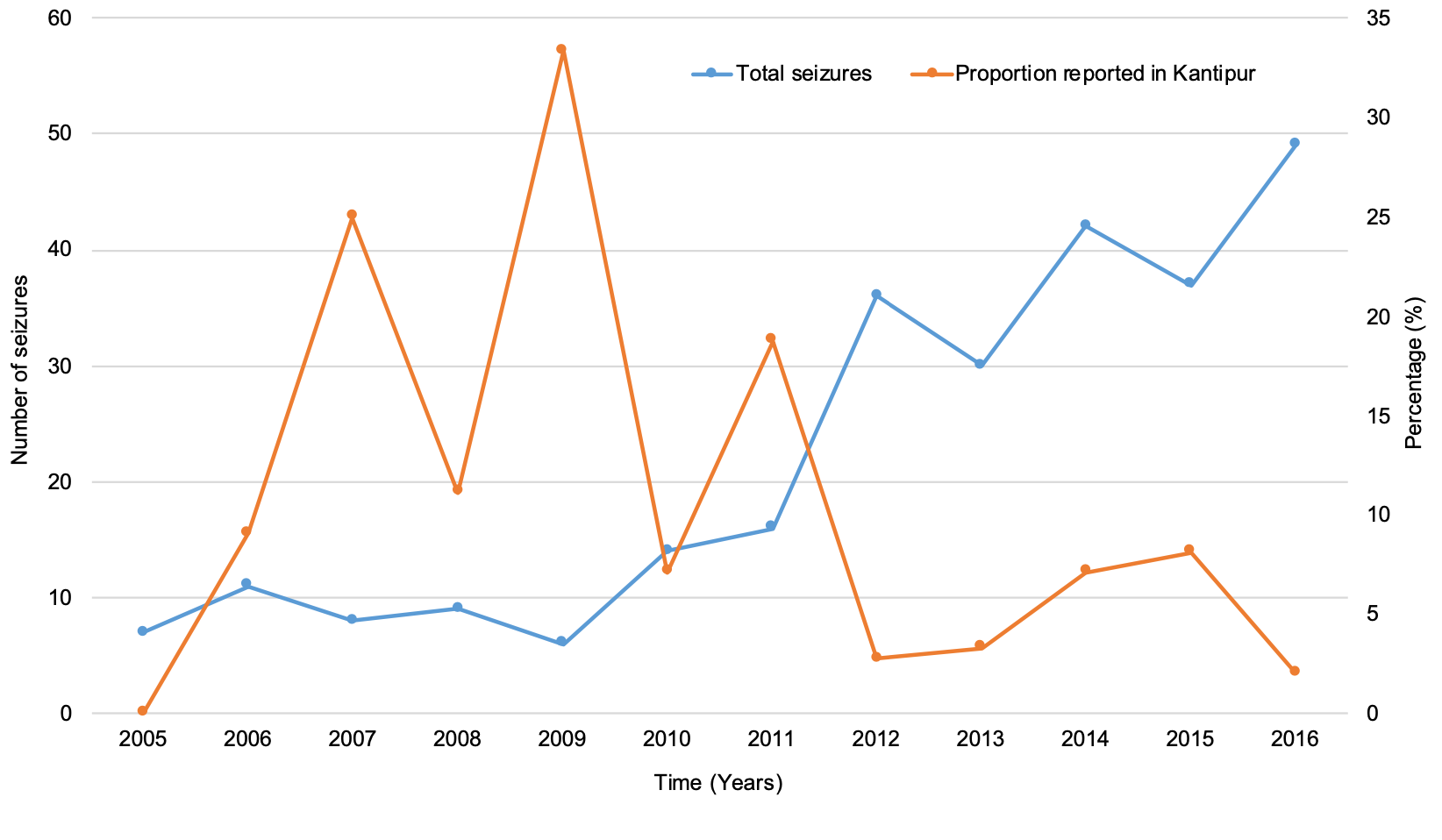
**

**Figure S4:** Yearly number of wildlife seizures in total and proportion reported by Kantipur for Kathmandu district only.


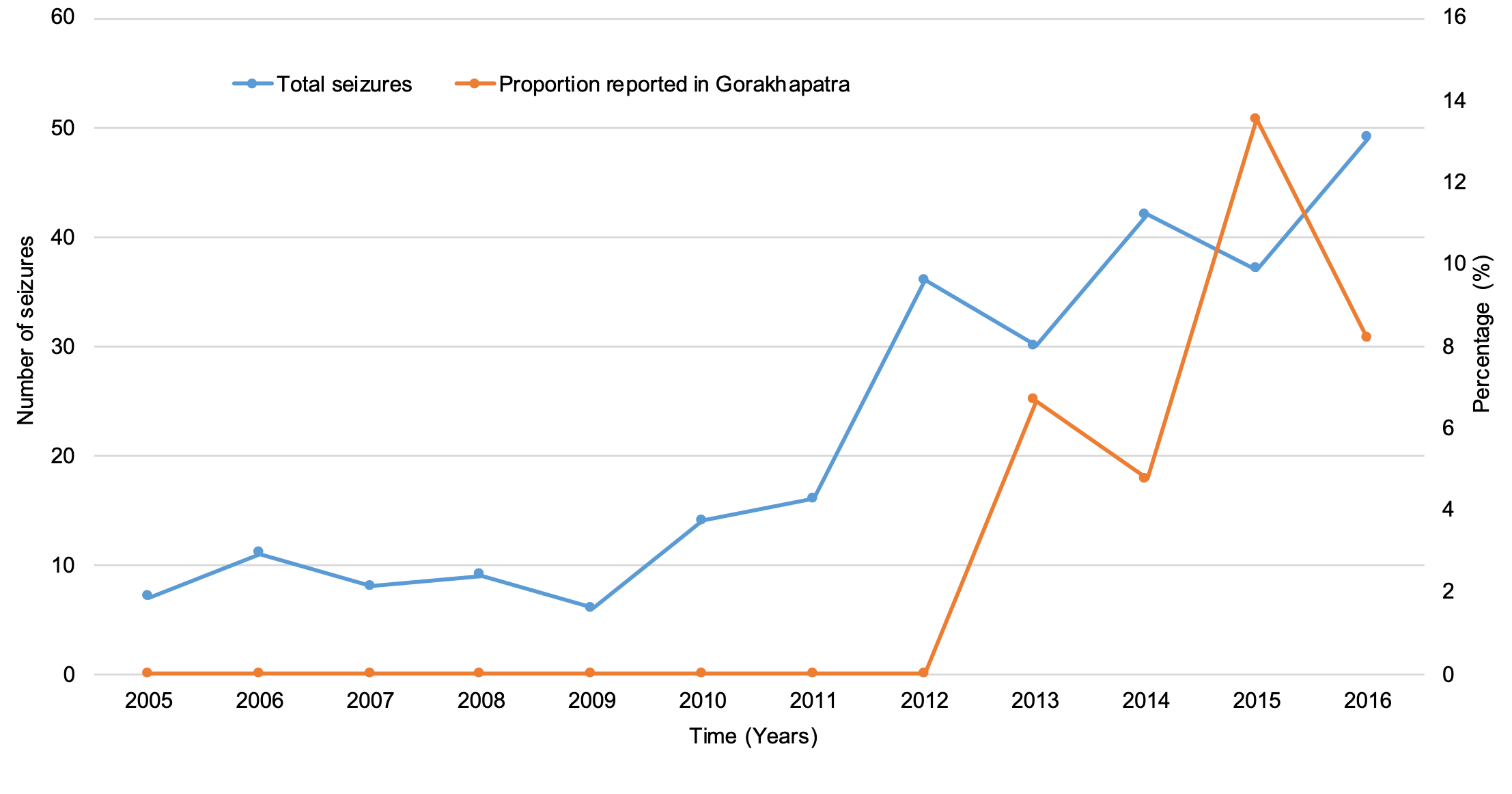


**Figure S5:** Yearly number of wildlife seizures in total and proportion reported by Gorkhapatra for Kathmandu district only.
